# Chemotherapy in Neuroendocrine Tumors

**DOI:** 10.3390/cancers13194872

**Published:** 2021-09-29

**Authors:** Satya Das, Taymeyah Al-Toubah, Jonathan Strosberg

**Affiliations:** 1 Department of Medicine, Division of Hematology and Oncology, Vanderbilt University Medical Center, Nashville, TN 37209, USA; satya.das@vumc.org; 2 Moffitt Cancer Center, Department of Gastrointestinal Oncology, Tampa, FL 33612, USA; taymeyah.al-toubah@moffitt.org

**Keywords:** chemotherapy, neuroendocrine tumors, platinum agents, alkylating agents, chemosensitivity

## Abstract

**Simple Summary:**

Cytotoxic chemotherapy is a standard therapy for patients with poorly differentiated neuroendocrine carcinomas, however, its role in patients with well differentiated neuroendocrine tumors is less defined. In this review, we describe the data supporting many of the chemotherapy regimens which have been tested in patients with well differentiated neuroendocrine tumors, specifically focusing on the impact of tumor features (e.g., primary tumor origin, grade and DNA damage repair defects) on chemosensitivity, and discuss future directions for chemotherapy as a combinatorial treatment modality for patients with this disease.

**Abstract:**

The role for cytotoxic chemotherapy in patients with well-differentiated neuroendocrine tumors (NETs) remains debated. Compared to patients with poorly differentiated neuroendocrine carcinomas (NECs) where chemotherapy is utilized ubiquitously, chemotherapy may play a more select role in patients with certain types of NETs (e.g., pancreatic tumors, higher grade tumors, and tumors possessing DNA damage repair defects). The primary types of chemotherapy that have been tested in patients with NETs include alkylating agent- and platinum agent-based combinations. Across regimens, chemotherapy appears to elicit greater antitumor activity in patients with pancreatic or grade 3 NETs. The role for chemotherapy in lower grade extra-pancreatic NETs remains undefined. Furthermore, while chemotherapy has demonstrated clinically meaningful benefit for patients in the systemic setting, its role in the adjuvant or neoadjuvant setting is as-of-yet undetermined. Finally, efforts to combine chemotherapy with targeted therapy and peptide receptor radionuclide therapy are ongoing, in hopes of improving the cytoreductive treatment options for patients with NETs.

## 1. Introduction

Neuroendocrine neoplasms (NENs) are heterogenous tumors whose incidence and prevalence continues to rise steadily [1]. Most NENs are well-differentiated neuroendocrine tumors (NETs), while the minority are poorly differentiated neuroendocrine carcinomas (NECs) [2,3]. Targeted therapies and somatostatin receptor-focused treatments are a staple of systemic therapy for patients with NETs, while cytotoxic chemotherapy is an established systemic treatment for NECs [4,5,6,7,8,9]. The role for chemotherapy in the treatment of NETs is much more nuanced and relevant for select tumor subsets. One reason the role for chemotherapy in NETs is less defined is that these tumors are often slowly proliferating; chemotherapy tends to be most effective against rapidly proliferating malignant cells [10]. Furthermore, the chemotherapy regimens that seem to demonstrate antitumor activity in NETs are different from the chemotherapy regimens (e.g., platinum etoposide doublet and platinum topoisomerase 1 inhibitor doublet) utilized for NECs [11,12,13,14]. In this review, we describe the data behind the chemotherapy regimens that have been tested in the systemic setting in patients with NETs, specifically focusing on the impact of tumor features (e.g., primary tumor origin, grade, and DNA damage repair defects) on chemosensitivity. We also discuss the possible role for chemotherapy in the adjuvant or neoadjuvant setting and as a combinatorial addition to existing systemic therapies for patients with this disease.

## 2. Literature Review

The PubMed database was queried with the search terms “chemotherapy for neuroendocrine tumors” or “chemotherapy in neuroendocrine tumors” to identify studies of chemotherapy in NETs. The search findings were limited to full-text articles published within the last 10 years with the understanding that important publications outside of this window would be captured by the included review articles. Identified studies that included only patients with poorly differentiated NECs were excluded. The reference listings from the key review articles identified were perused to identify other publications that may not have been identified from the original search. The American Society of Clinical Oncology Meeting Library was also queried using the following search terms “FOLFOX neuroendocrine tumors,” “CAPOX neuroendocrine tumors,” “CAPTEM neuroendocrine tumors,” “alkylating therapy neuroendocrine tumors,” “platinum chemotherapy neuroendocrine tumors”, and “chemotherapy neuroendocrine tumors” to identify unpublished studies describing the experience of chemotherapy in patients with NETs. The search was limited to abstracts published within the last 10 years. Identified studies that included only patients with poorly differentiated NECs were excluded.

## 3. Current Evidence for Chemotherapy in the Systemic Setting

### 3.1. Alkylating Agents

#### 3.1.1. Streptozocin-Based Regimens

##### Pancreatic NETs

Streptozocin is an antibiotic derived from the Gram-positive bacterium Streptomyces, which demonstrated toxicity against pancreatic cells in animal models [15]. The initial studies with streptozocin in patients with pancreatic NETs demonstrated antitumor activity; however, interpreting these studies is challenging given the outdated response criteria that was used for tumor assessments and a significant nausea and vomiting burden patients experienced (as the trials preceded the advent of modern antiemetics) [16]. Combination therapy with streptozocin plus fluorouracil became the treatment standard for patients with advanced pancreatic NETs after demonstrating superior antitumor activity compared to streptozocin monotherapy.

More than a decade later, streptozocin plus doxorubicin was compared against streptozocin plus fluorouracil [17]. In this study, 105 patients with metastatic or unresectable pancreatic NETs were randomized in a 1:1:1 fashion to streptozocin plus fluorouracil (500 mg/m^2^ intravenously (IV) D1-D5 for each drug every 6 weeks), streptozocin plus doxorubicin (streptozocin 500 mg/m^2^ IV D1-D5 and doxorubicin 50 mg/m^2^ IV D1 and D22 every 6 weeks) or chlorozotocin (150 mg/m^2^ IV D1 every 7 weeks). The ORR was superior with streptozocin plus doxorubicin compared to streptozocin plus fluorouracil (69% vs. 45%, *p* = 0.05) and chlorozotocin (69% vs. 30%, *p* = 0.002). The median OS in patients treated with streptozocin plus doxorubicin was significantly longer than patients treated with streptozocin plus fluorouracil or chlorozotocin (2.2 years vs. 1.4 years vs. 1.5 years, *p*-value not provided). With regards to adverse events, more than 80% of patients in both streptozocin combination arms experienced vomiting.

Despite the cytotoxicity of streptozocin-based regimens, the regimens were much less-favored compared to other treatments until the advent of modern antiemetics. Several ongoing studies are exploring streptozocin combinations in patients with pancreatic NETs. The SEQTOR study (NCT02246127) is a randomized phase III study that is assessing whether streptozocin plus fluorouracil followed by everolimus upon progression or everolimus followed by streptozocin plus fluorouracil upon progression is the optimal sequence in patients with pancreatic NETs. The BETTER-2 study (NCT03351296) is a randomized phase II study comparing streptozocin plus fluorouracil +- bevacizumab or capecitabine plus temozolomide +- bevacizumab in patients with pancreatic NETs.

##### Extra-Pancreatic NETs

Streptozocin-based regimens have also been tested in patients with extra-pancreatic NETs, which were (and, at times, still are) referred to as carcinoid tumors. A summary of several studies with the agent in patients with extra-pancreatic NETs is presented in Table 1. Relative to the results of studies presented in patients with pancreatic NETs, patients with extra-pancreatic NETs treated with streptozocin-based regimens demonstrated lower ORR and shorter survival times.

#### 3.1.2. Dacarbazine-Based Regimens

##### Pancreatic NETs

Small retrospective experiences suggested the antitumor activity of dacarbazine in patients with advanced pretreated predominantly pancreatic NETs [23,24]. Based upon this activity, patients with pancreatic NETs were treated with dacarbazine (850 mg/m^2^ IV D1 every 4 weeks) in the phase II E6282 trial [25]. Of the 50 response eligible patients, 28 (56%) were systemic therapy naïve. The ORR (by the WHO criteria) was 33%, however, differed based upon the prior treatment received. The ORR in treatment-naïve patients and pretreated patients was 50% and 13.6%, respectively. The median response duration in patients was 10 months, and the median OS was 19.3 months. A total of 16 patients (30%) experienced grade 3/4 toxicities, 5 patients experienced grade 4 hematologic toxicities, 3 experienced grade 4 nonhematologic toxicities, and 7 patients experienced grade 3 vomiting.

A larger retrospective study analyzed the outcome of 50 patients with pancreatic NETs treated with dacarbazine (650 mg/m^2^ IV D1 every 4 weeks) [26]. Of this cohort, 32% achieved a partial response. The median progression-free survival (PFS) of patients with pancreatic NETs was 10 months, while it was 6 months for patients with nonpancreatic NETs (*p* = 0.65).

Dacarbazine is a prodrug that shares the same active metabolite (5-(3-methyltriazen-1-yl) with temozolomide [27]. Given the oral administration and better tolerability of temozolomide, this agent has replaced dacarbazine as a treatment option for patients with NETs. Although the efficacy of dacarbazine vs. temozolomide-based regimens has not been compared in the context of a trial, the two regimens were compared in a retrospective study [28]. A total of 247 patients (82.3% possessed pancreatic NETs, 78.9% with grade 1/2 NETs) were treated with either dacarbazine plus fluorouracil or capecitabine plus temozolomide. The patient distribution was not balanced, as 94 and 153 patients were treated with the former and latter regimens, respectively. There was no difference in the ORR (38.3% vs. 39.2%, *p* = 0.59) or median PFS (*p* = 0.86). Interestingly, the temozolomide-based regimen was associated with higher rates of grade 3/4 adverse events compared to the dacarbazine-based regimen (24.7% vs. 8.5%, *p* = 0.002), as well as a higher risk of progression (hazard ratio (HR) 1.65, *p* = 0.004). While there were inherent limitations to this analysis, it does suggest that dacarbazine-based combination regimens may have similar activity to temozolomide-based regimens, particularly in patients with pancreatic NETs.

##### Extra-Pancreatic NETs

A small prospective phase II study tested dacarbazine (250 mg/m^2^ IV D1-D5 every 4 to 5 weeks) in patients with extra-pancreatic NETs [29]. The median time to tumor progression was 4.5 months, while the median OS was 11.75 months. In a subsequent phase II study, dacarbazine (400 mg/m^2^ IV D1 and D2) was combined with fluorouracil (1 g/m^2^ IV D1 and D2) and leucovorin (200 mg/m^2^ IV D1 and D2) in 21-day cycles [30]. Of the 18 patients, nine possessed nonpancreatic NETs; only one patient experienced a partial response. The efficacy of the treatment was deemed to be insufficient in patients with nonpancreatic NETs.

#### 3.1.3. Temozolomide-Based Regimens

Temozolomide is an oral alkylating agent with improved blood–brain barrier penetration compared to other agents of its class [31]. The agent was tested in the monotherapy setting in an unselected group patients with well-differentiated NETs [32]. In this retrospective study of 36 patients with metastatic or unresectable disease, the patients received temozolomide (150–200 per os (PO) mg/m^2^ D1-D5 every 28 days); 24 patients possessed extra-pancreatic NETs, while 12 patients possessed pancreatic NETs. Of these patients, 15% (four of the five responders possessed lung NETs) experienced a PR. The median time to progression was 7 months for all patients. No significant difference in the antitumor response was observed between patients with O^6^-methylguanine-DNA methyltransferase (MGMT) deficiency or intact MGMT expression. The patients experienced grade 3/4 thrombocytopenia, fatigue, and anemia in 14%, 6%, and 3% of the cases, respectively. No cases of grade 3/4 nausea or vomiting were observed.

A subsequent prospective study tested the combination of temozolomide (150 mg/m^2^ PO D1-D7 every other week) and thalidomide (50–400 mg PO daily) in 29 patients with advanced NETs [33]. The primary endpoint of the study was the ORR. Of the treated patients, 15 (52%) possessed extra-pancreatic NETs, 11 (38%) possessed pancreatic NETs, and 3 (10%) possessed parangliomas/pheochromocytomas. The ORR for all the patients was 25%, with five of the seven responding patients possessing pancreatic NETs. The median duration of the response was 13.5 months, and the median PFS was not reached. Grade 3/4 adverse events were quite rare, with 7, 7 6, 6, and 3% of patients experiencing anemia, fatigue, neutropenia, nausea/vomiting, and thrombocytopenia.

The combination of temozolomide (150 mg/m^2^ PO D1-D7 and D15-D21) plus bevacizumab (5 mg/kg IV D1 and D15) every 28 days was tested in a phase II study in patients with advanced NETs [34]. The primary endpoint of the study was the ORR. A total of 34 patients (56% with extra-pancreatic NETs and 44% with pancreatic NETs) were treated. The ORR of the entire cohort was 15% with all the responses occurring in patients with pancreatic NETs. The median PFS for all the patients was 11 months, with a median PFS of 14.3 months for the patients with pancreatic NETs and 7.3 months in the patients with extra-pancreatic NETs. Grade 3/4 thrombocytopenia, nausea/vomiting, fatigue, neutropenia, and proteinuria were observed in 18, 15, 6, 6, and 3% of the patients, respectively. Based on the signal of the activity seen in pancreatic NETs, other temozolomide-based combinations were explored in this tumor group.

**Figure 1:**
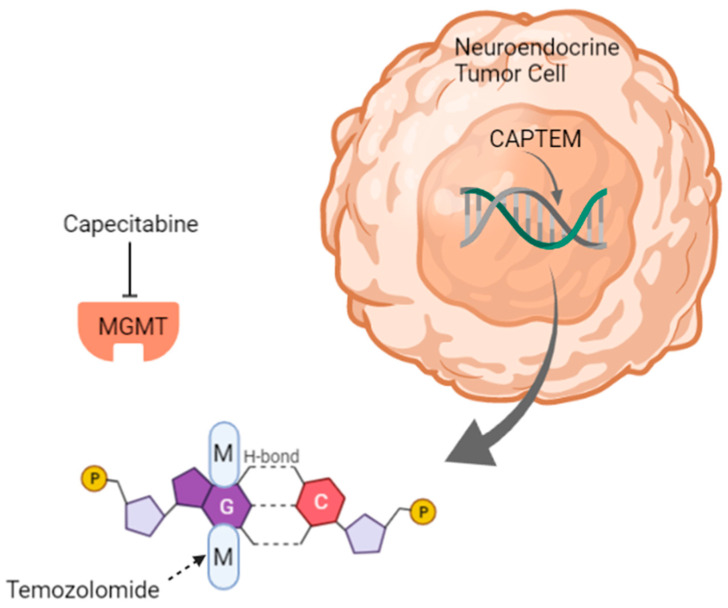
A depiction of the mechanism of action of capecitabine plus temozolomide in a neuroendocrine tumor. Temozolomide adds a methyl group to the O^6^ position of guanine, resulting in mismatched bases and cell death. The MGMT enzyme typically transfers methyl groups from the O^6^ position of guanine to its cysteine residue; however, this is inhibited by capecitabine. The figure was created in Biorender. Abbreviations: M, methyl group; CAPTEM, capecitabine plus temozolomide; G, guanine; C, cytosine.

The combination of capecitabine plus temozolomide is perhaps the temozolomide combination with the strongest preclinical rationale. Capecitabine, along with other fluoropyrimidines, is thought to deplete MGMT, the enzyme responsible for repairing the DNA damage created by temozolomide [35] (Figure 1).

The largest retrospective experience to date in patients with well-differentiated NETs treated with capecitabine plus temozolomide was recently published [36]. From a total of 462 patients treated with capecitabine plus temozolomide between 2008 and 2019, 79% possessed well-differentiated NETs. Patients with pancreatic NETs comprised the majority (71.4%) of the included patients in the analysis. The ORR was significantly longer in patients with pancreatic NETs compared to those with extra-pancreatic NETs (51.5% vs. 31.8%, *p* < 0.001). The median PFS (23 months vs. 10 months, *p* < 0.001) and OS (62 vs. 28 months, *p* < 0.001) were significantly longer in patients with pancreatic NETs compared to those with extra-pancreatic NETs. Grade 3/4 thrombocytopenia, neutropenia, and lymphopenia were observed in 7%, 3%, and 2% of the patients, respectively. A total of three patients, all who received prior peptide receptor radionuclide therapy (PRRT), developed myelodysplastic syndrome (MDS).

##### Pancreatic NETs

A retrospective analysis explored the antitumor activity of capecitabine (750 mg/m^2^ PO BID D1-D14) plus temozolomide (200 mg/m^2^ PO QD D10-D14) every 28 days in chemotherapy-naïve patients with pancreatic NETs [37]. Ondansetron was taken before temozolomide. Among the 30 treated patients, 83% possessed grade 1/2 tumors. The documented ORR was 70%. The median PFS was 18 months, while the median OS was not reached. With regards to toxicity, only 12% of the patients experienced grade 3/4 adverse events. Another large retrospective analysis explored the activity of capecitabine (750 mg/m^2^ PO BID D1-D14) plus temozolomide (150–200 mg/m^2^ PO QD D10-D14) in 100 patients with well-differentiated advanced pancreatic NETs (median Ki-67 11%) [38]. The patients received a mean number of 7 ± 4.5 cycles with a median PFS of 21.4 months. The ORR was 51%, and the median OS in the patients treated with the combination was 75.2 months.

A prospective phase II study assessed the activity of capecitabine (750 mg/m^2^ PO BID D1-D14) plus temozolomide (150–200 PO QD mg/m^2^ D10-D14) every 28 days in patients with well-differentiated NETs [39]. Of the 11 patients with pancreatic NETs, the ORR and DCR were 36 and 91%, respectively. The median PFS for these patients was >20 months, while the median OS was >24.4 months. ECOG 2211 was a randomized phase II study that compared the antitumor activity of capecitabine (750 mg/m^2^ PO BID D1-D14) plus temozolomide (200 mg/m^2^ PO QD D10-D14) vs. temozolomide (200 mg/m^2^ PO QD D1-D5) monotherapy in 144 patients with advanced grade 1/2 pancreatic NETs [40]. The primary endpoint of the study was PFS. The median PFS was 22.7 months in the combination arm vs. 14.4 months in the temozolomide monotherapy arm (HR 0.58, *p* = 0.02). The median OS was not reached in the combination arm vs. 38 months in the temozolomide monotherapy arm (HR 0.41, *p* = 0.012). No significant difference in the ORR was observed between the two arms (*p* = 0.47). No difference in the PFS or OS was observed based upon the tumor grade, suggesting that patients with both grade 1 and grade 2 tumors benefited. With regards to toxicity, 44% of the patients in the combination arm experienced grade 3/4 adverse events compared to 22% of the patients in the temozolomide monotherapy arm (*p* = 0.007). The most common grade 3/4 adverse events in the combination arm were neutropenia (13%), thrombocytopenia (8%), nausea (8%), vomiting (8%), and diarrhea (8%). The correlative endpoints of exploring the outcomes in patients based upon MGMT expression (by immunohistochemistry and promoter methylation) remain pending.

Prospective studies assessing the optimal duration of therapy for capecitabine plus temozolomide have not been conducted. The retrospective analyses done to data have reported heterogenous findings. Several studies suggested that the maximal time to response occurs within 4–6 months of starting therapy [36]; however, whether to continue capecitabine plus temozolomide indefinitely or for a finite period of time varies based on the practice patterns of individual NET specialists.

##### Extra-Pancreatic NETs

A number of retrospective analyses have assessed the activity of capecitabine plus temozolomide in patients with extra-pancreatic NETs and are summarized in Table 2.

Of these studies, the ones focusing specifically on patients with lung NETs are discussed in the following paragraphs. A study by Al-Toubah et al. explored the activity of capecitabine (750 mg/m^2^ PO BID D1-D14) plus temozolomide (200 mg/m^2^ PO QD D10-D14) in patients with lung NETs [43]. The average starting dose of capecitabine was 558 mg/m^2^ BID, while the average starting dose of temozolomide was 170 mg/m^2^ after the dose modifications. Of the 20 included patients, 70% possessed typical lung NETs, 25% possessed atypical lung NETs, and 5% possessed an undefined tumor differentiation pattern. The ORR and DCR in the patients were 30 and 85%, respectively. The median PFS and OS in the patients were 13 and 68 months, respectively. Only one instance of grade 3 abdominal pain and two instances of grade 4 thrombocytopenia were noted.

Another study explored the activity of capecitabine (750 mg/m^2^ PO BID D1-D14) plus temozolomide (200 mg/m^2^ PO QD D10-D14) in patients with lung NETs [44]. In this study of 33 patients, 61% possessed atypical lung NETs, 30% typical lung NETs, and 9% possessed a lung NET NOS. The ORR was 18.2%, the median PFS was 9 months, and the median OS was 30.4 months. The results from both these studies suggest a role for capecitabine plus temozolomide in patients with lung NETs.

From the collection of studies presented and others published in the existing literature [46], it appears that capecitabine plus temozolomide appears to elicit greater tumor cytoreduction and more prolonged tumor suppression in pancreatic NETs compared to nonpancreatic NETs. There are no studies with the regimen indicating an appreciable objective response rate in midgut NETs.

##### Outcomes Based upon MGMT Status

The MGMT enzyme is responsible for repairing the DNA damage from temozolomide (and other alkylating agents) primarily by removing the methyl group from the O^6^ position, which restores the DNA of affected cells into a normal conformation [47]. Based upon this mechanistic rationale, it has been hypothesized that patients with NETs with MGMT deficiency should be more sensitive to alkylating agent-based therapy compared to patients with NETs with intact MGMT. The clinical data supporting this hypothesis have been inconsistent. Some of the studies demonstrating contrasting results between MGMT expression and the alkylating agent-based therapy response are discussed in the subsequent sections.

A retrospective analysis assessed MGMT expression (by immunohistochemistry) in archival NET specimens and compared the prevalence of MGMT deficiency in pancreatic NETs and extra-pancreatic NETs [48]. Of the 37 pancreatic NET specimens, 51% were MGMT-deficient, while, among the 60 extra-pancreatic NET specimens, 0% were MGMT-deficient. In a second part of the study, the treatment outcomes in NET patients who had previously received temozolomide were assessed based upon the prevalence of MGMT loss from archival tissue. Among 21 patients with archival tissue available, five patients possessed tumors with MGMT deficiency while 16 patients possessed tumors with intact MGMT expression. Patients with MGMT-deficient tumors demonstrated a significantly improved ORR compared to patients with tumors with intact MGMT expression (80% vs. 0%, *p* = 0.001). Patients with MGMT-deficient tumors also demonstrated improved median PFS (19.2 months vs. 9.3 months) and median OS (NR vs. 19.1 months) compared to patients with tumors with intact MGMT expression, though these differences were not statistically significant.

Other studies exploring the association between MGMT deficiency in tumors and temozolomide responsiveness have assessed the MGMT expression status by promoter methylation assays [49,50]. The larger retrospective analysis assessed the outcomes in 95 patients with NETs treated with temozolomide-based regimen. Of these patients, 43, 30, and 22 patients possessed pancreatic, other, and lung primary tumors, respectively. Twenty-eight patients possessed tumors with MGMT promoter methylation (expression loss), while 67 patients possessed tumors without promoter methylation (expression intact). Patients with tumors with MGMT expression loss experienced a significantly improved ORR (51.8% vs. 17.7%, *p* = 0.001) and PFS (21 months vs. 8 months, *p* = 0.017) compared to patients with intact MGMT expression.

A large retrospective analysis specifically assessed potential biomarkers of the response for capecitabine plus temozolomide in 143 patients with pancreatic NETs [51]. Of these patients, 115 possessed grade 1/2 tumors, and 52 possessed archival tissue available for MGMT expression by immunohistochemistry. A total of 15 patients possessed MGMT-deficient tumors while 37 patients possessed MGMT expression intact tumors. No difference in the ORR (*p* = 0.66), PFS (*p* = 0.25), or OS (*p* = 0.4) was observed in patients based upon the tumor MGMT status. At this juncture, the pending correlative analyses from the prospective randomized phase II ECOG 2211 study may best reveal the true association between the tumor MGMT expression status and response to temozolomide.

##### Other Candidate DNA Damage Repair Defects

Apart from MGMT deficiency, no other DNA damage repair defects have been targeted clinically in patients with NETs. Some other possible candidates include the tumor suppressor PTEN and mismatch repair proteins (MLH1, MSH2, MSH6, and PMS2). PTEN, apart from its role in controlling cellular proliferation and apoptosis, stimulates several pathways of DNA repair, including homologous recombination, nonhomologous end joining, and nucleotide excision repair [52]. Preclinical studies in other tumor types such as glioblastoma suggest that PTEN deficiency may sensitize tumors to temozolomide and other alkylating agents [53]. Mismatch repair (MMR) deficiency can occur through a variety of mechanisms, including promoter methylation and loss of expression. Several retrospective tissue-based studies have suggested that pancreatic NETs demonstrate MMR deficiency in 15–36% of cases [54,55]. Whether MMR deficiency may predict chemosensitivity in NETs remains to be determined. From other tumor types, while it is associated with immunotherapy responsiveness, it has been associated with the chemotherapy refractory status [56,57].

### 3.2. Platinum Agents

#### 3.2.1. Oxaliplatin Based-Regimens

Oxaliplatin, rather than cisplatin or carboplatin, is the platinum agent that has demonstrated meaningful activity in patients with well-differentiated NETs.

**Figure 2:**
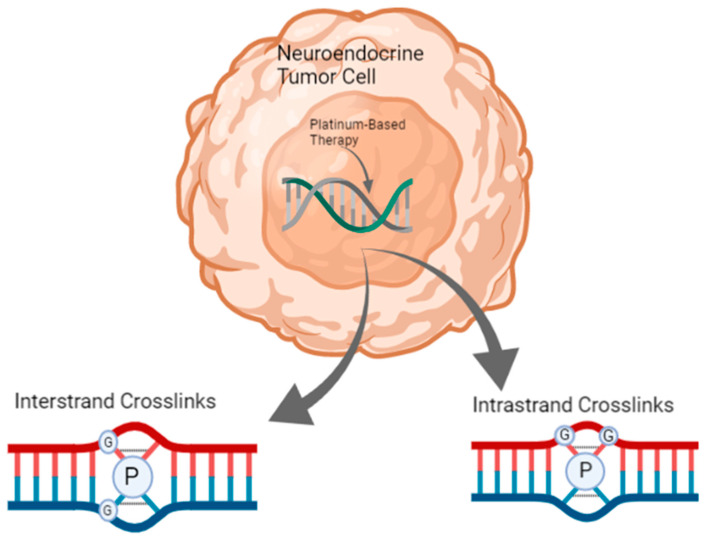
A depiction of the mechanism of action of platinum-based therapies (e.g., oxaliplatin, cisplatin, and carboplatin) in a neuroendocrine tumor. The platinum agent binds to the N^7^ position of guanine to form inter-strand or intra-strand crosslinks, which stall DNA replication and cause cell death. The table was created in Biorender. Abbreviations: P, platinum agent; G, guanine.

The oxaliplatin combination regimen that has been most tested in patients with NETs is fluorouracil plus oxaliplatin (FOLFOX) or capecitabine plus oxaliplatin (CAPOX). The DNA-damaging mechanism of action of platinum agents is described in Figure 2.

##### Pancreatic NETs

A retrospective single-institution analysis explored the antitumor activity of FOLFOX (unspecified dose schedule) in 31 patients with pancreatic NET that progressed on prior therapy with capecitabine plus temozolomide [58]. Of these patients, 25 received FOLFOX, while six received FOLFOX plus bevacizumab, an anti-vascular endothelial growth factor (VEGF)-A monoclonal antibody. The patients were heavily pretreated with a median of three prior treatment lines. With regards to the tumor grade, 61, 19, and 20% possessed grade 1/2, grade 3, and not otherwise defined tumors, respectively. The ORR was 45%, the median PFS was 6 months, and the median OS was 16 months (from the study treatment onset) in the patients. No statistically significant difference in the outcomes was observed between patients who received FOLFOX alone or in combination with bevacizumab. With regards to toxicities, grade 3 neuropathy, hepatic encephalopathy, neutropenia, fatigue, and hypoglycemia occurred in 6.5, 6.5, 3, 3, and 3% of the patients, respectively.

Another retrospective analysis explored the antitumor activity of FOLFOX (modified FOLFOX-6 regimen with oxaliplatin 85 mg/m^2^ IV D1, leucovorin 100 mg/m^2^ IV D1, bolus fluorouracil 400 mg/m^2^ IV D1, and fluorouracil 2400 mg/m^2^ IV as a continuous infusion over 46 h every 14 days) in 48 patients with advanced well-differentiated NETs [59]. Of these patients, 33 possessed pancreatic NETs. The patients possessed grade 1/2 and grade 3 tumors in 69 and 31% of the cases, respectively. Of the patients with pancreatic NETs, 61% were treated with FOLFOX in the first-line setting. The patients received a median of six cycles of treatment with an ORR of 33%, DCR of 81.8%, median PFS of 12.6 months (total population not separated by the primary tumor site), and median OS of 29.4 months (total population not separated by the primary tumor site).

The largest retrospective experience of patients with well-differentiated NETs treated with FOLFOX reported to date included 88 patients with pancreatic NETs [60]. The ORR in these patients was 31%, the median PFS was 9 months, and the median OS was 30 months. Among the patients with pancreatic NETs, those with insulinomas demonstrated the longest median PFS (22 months).

There was a pooled analysis of two prospective phase II studies of FOLFOX (modified FOLFOX-6 regimen) plus bevacizumab (5 mg/kg IV D1) every 14 days or CAPOX (oxaliplatin 130 mg/m^2^ IV D1 and capecitabine 850 mg/m^2^ PO BID D1-D14) plus bevacizumab (7.5 mg/kg IV D1) every 21 days in patients with NETs [61]. Of the 36 patients in the FOLFOX trial, 12 possessed pancreatic NETs. The ORR in these patients was 41.7%, and the DCR was 100%. Of the 40 patients in the CAPOX trial, 16 possessed pancreatic NETs. The ORR was 18.8%, while the DCR was 87.5%. The median PFS of the patients with pancreatic NETs in the FOLFOX and CAPOX arms was 21 months and 15.7 months, respectively. The median OS of the patients with pancreatic NETs in the FOLFOX and CAPOX arms was 31 and 38 months, respectively.

##### Extra-Pancreatic NETs

In the aforementioned retrospective experience, 37, 13, 7, and 4 patients with small intestinal, unknown primary, gastric, and rectal NETs, respectively, were treated with FOLFOX [60]. The ORR in the patients with small intestinal, unknown primary, gastric, and rectal NETs was 13, 38, 14, and 25%, respectively. The median PFS in the patients with small intestinal, unknown primary, gastric, and rectal NETs was 9 months, 6 months, 14 months, and 4 months, respectively. The median OS in the patients with small intestinal, unknown primary, gastric, and rectal NETs was 28 months, 15 months, 31 months, and 25 months, respectively.

The patients with extra-pancreatic NETs from the FOLFOX or CAPOX plus bevacizumab prospective studies demonstrated the following outcomes [61]. Of the 36 patients in the FOLFOX trial, 22 possessed extra-pancreatic NETs. The ORR in these patients was 18.2%, while the DCR was 90.9%. Of the 40 patients in the CAPOX trial, 20 possessed extra-pancreatic NETs. The ORR in these patients was 5%, while the DCR was 65%. The median PFS of the patients with extra-pancreatic NETs in the FOLFOX and CAPOX arms was 19.3 months and 19.1 months, respectively. The median OS of the patients with extra-pancreatic NETs in the FOLFOX and CAPOX arms was 33.1 and 42.2 months, respectively.

Oxaliplatin-based regimens appear to elicit less tumor cytoreduction in nonpancreatic NETs compared to pancreatic NETs. The duration of the antitumor response, however, appears to be more similar between the tumor types than seen with alkylating agent-based regimens.

### 3.3. Chemotherapy in Grade 3 NETs

#### 3.3.1. Cisplatin or Carboplatin-Based Regimens

Grade 3 well-differentiated NETs are a relatively new pathologic entity that only recently were defined as a separate tumor category [62,63]. The majority of grade 3 NETs arise in the pancreas, stomach, or colorectum [64]. Given the heterogeneous behavior of these tumors, a significant degree of uncertainty exists about the optimal chemotherapy regimens for this tumor type. Platinum doublet chemotherapy with cisplatin or carboplatin plus etoposide is a staple of therapy for the patients with poorly differentiated grade 3 NECs; however, the activity of the regimen(s) in patients with well-differentiated NETs is less clear [11,12,64,65,66,67,68]. An ongoing randomized phase II study is comparing platinum etoposide doublet chemotherapy vs. capecitabine plus temozolomide in patients with grade 3 NENs (including NETs and non-small cell NECs) (NCT02595424). This trial may answer whether platinum-based chemotherapy regimens are most active in patients with NECs or may play a role in the treatment of patients with NETs as well. Currently, there is more data for alkylating agents or oxaliplatin-based regimens in patients with grade 3 NETs. The data behind these regimens will be discussed in the following sections.

#### 3.3.2. Alkylating Agent-Based Regimens

A retrospective analysis explored the outcomes in patients with grade 3 pancreatic NENs [69]. Of the 45 included patients, 16 possessed well-differentiated NETs. The ORR and DCR in the patients with NETs treated with alkylating agents was 50 and 75%, respectively. The ORR and DCR in the patients with NETs treated with platinum-based therapy was 10 and 60%, respectively.

Another retrospective study explored the activity of capecitabine plus temozolomide in patients with grade 3 NENs [69]. Of the 32 included patients, 20 possessed gastroenteropancreatic NETs (Ki-67 index range 20–54%), while 12 possessed gastroenteropancreatic NECs (median Ki-67 index ≥ 55%). The predominant primary tumor locations for the patients were the pancreas (40.6%), colorectum (18.7%), and stomach (15.6%). The majority (75%) of patients received the therapy in the second-line and beyond setting. The median PFS in the NET group was significantly higher than in the NEC group (15.3 months vs. 3.3 months, HR 0.18, *p* < 0.001). The median OS in the NET group was also significantly higher in the NET group than in the NEC group (22 months vs. 4.6 months, HR 0.25, *p* = 0.0178). The ORR and DCR in the patients with NETs treated with the regimen was 50% and 70%, respectively. The ORR and DCR in the patients with NECS treated with the regimen was 8.3% and 33%, respectively.

A large multicenter retrospective analysis explored the activity of temozolomide-based regimens (92% capecitabine plus temozolomide and 8% temozolomide) in the patients with grade 3 gastroenteropancreatic NENs (75% of the pancreatic primary origin) [70]. Of the 118 patients (75% with pancreatic primary tumors), 56 possessed well-differentiated NETs, 21 possessed tumors of undefined histology, and 41 possessed poorly differentiated NECs. The patients treated with capecitabine plus temozolomide vs. temozolomide monotherapy had a significantly longer time to treatment failure (TTF) (5.7 months vs. 1 month, *p* = 0.02). the patients with well-differentiated NETs had a significantly improved ORR compared to the patients with poorly differentiated NECs (52% vs. 26%, *p* = 0.02). The patients with well-differentiated NETs also experienced a significantly longer OS compared to the patients with poorly differentiated NECs (30.1 months vs. 12.8 months, *p* = 0.008).

A prospective phase II trial explored the activity of capecitabine plus temozolomide in the patients with grade 3 gastroenteropancreatic NENs (Ki-67 < 55%) (43.3% pancreatic primary origin) [71]. Of the 30 patients in the full analysis, seven were noted by a post hoc central pathology review to actually possess grade 3 NECs. The ORR was 30%, while the DCR was 76.7%. The ORR varied by the Ki-67 index, as patients with a Ki-67 < 30%, Ki-67 30–55%, and >55% experienced ORRs of 18.2%, 50%, and 0%, respectively. The median PFS was 5.9 months, and the median OS was NR. The PFS and OS also varied by the Ki-67 index, as patients with a Ki-67 index < 30% demonstrated a median PFS and OS of 16.5 months and NR, respectively, patients with a Ki-67 index of 30–55% experienced a median PFS and OS of 6.8 months and NR, respectively, and patients with a Ki-67 index > 55% experienced a median PFS and OS of 2.7 months and 5.7 months, respectively. The median PFS was longer in patients who received the regimen in a first-line setting compared to a later-line setting; however, this difference was not statistically significant (16.5 months vs. 4.6 months, *p* = 0.425).

#### 3.3.3. Oxaliplatin-Based Regimens

A retrospective multicenter analysis assessed the effectiveness of various first-line systemic therapies in 131 patients with grade 3 gastroenteropancreatic NETs (median Ki-67 30%; 70% with primary pancreatic tumors) [72]. Of the included patients, 36 received FOLFOX, 21 received temozolomide-based therapy, and 34 patients received platinum etoposide doublet therapy. The ORR and DCR was the highest for the patients who received FOLFOX at 52.8 and 80.6%, respectively. In comparison, the ORR and DCR were 28.6 and 66.7%, respectively, in patients who received temozolomide-based therapy. The median PFS in patients receiving first-line FOLFOX and temozolomide-based regimens was 6.2 months and 12 months, respectively. Of the included patients, 89 received second-line therapy. The median PFS in patients receiving second-line therapy with FOLFOX and temozolomide-based regimens was 13.9 months and 8.9 months, respectively. Though this study suggests that the optimal use of FOLFOX may be in the second-line setting for post-temozolomide-based therapy, prospective studies need to be conducted to determine the optimal chemotherapy sequencing.

## 4. Current Evidence for Chemotherapy in the Adjuvant or Neoadjuvant Setting

There is no prospective randomized data for chemotherapy in the adjuvant or neoadjuvant setting for patients with well-differentiated NETs. As such, many of the national NET guideline panels do not currently recommend chemotherapy in these settings for patients with well-differentiated NETs [73,74]. Several retrospective analyses have explored the role for adjuvant and neoadjuvant chemotherapy, predominantly in patients with localized pancreatic NETs, and demonstrated no clear benefit of either approach [75,76].

Other retrospective analyses suggest that neoadjuvant chemotherapy may play a role in patients with metastatic pancreatic NETs. The first of these was a single center analysis that assessed the outcomes in 67 patients with liver-limited pancreatic NETs who underwent R0/R1 resections [77]. Of these patients, 42 possessed grade 1/2 tumors, 4 patients possessed grade 3 disease, and 21 possessed diseases of unspecified grades. A total of 27 patients received neoadjuvant fluorouracil, doxorubicin, and streptozocin (fluorouracil 400 mg/m^2^ IV D1-D5, streptozocin 400 mg/mg IV D1-D5, and doxorubicin 40 mg/mg^2^ IV D1 every 28 days). The median number of neoadjuvant chemotherapy cycles administered was four. Among the patients who received neoadjuvant therapy, the ORR was 63%. Further, 18.5% of the patients initially felt to be unresectable were able to undergo surgical resection. While the patients treated with neoadjuvant therapy had a higher proportion of poor prognostic factors (bulkier disease and synchronous disease), the survival outcomes were the same as the outcomes in the patients treated with surgery alone. A more recent multicenter study assessed the outcomes in 30 patients with locally advanced or metastatic pancreatic NETs with resectable disease in the liver; 25 patients possessed grade 1/2 tumors, while five patients possessed grade 3 tumors [78]. The median number of capecitabine plus temozolomide cycles administered was four. The patients experienced an ORR of 43%, 5-year PFS of 30%, and 5-year OS of 63%.

Adjuvant prospective studies are in development, with one soon-to-be-activated trial exploring the role of capecitabine plus temozolomide in patients with resected pancreatic NETs. This remains an area of active clinical investigation.

## 5. Combinatorial Approaches with Chemotherapy

The number of targeted and theranostic options for patients with NETs have expanded significantly over the last decade; however, the cytoreductive capacity of these agents remains quite modest [4,5,6,7]. As such, investigators have sought to combine some of these treatments with chemotherapy to improve their antitumor effect. Several prospective combination studies are discussed in Table 3 and the subsequent sections.

### 5.1. Chemotherapy plus PRRT

Chemotherapy has been combined with PRRT with Lutetium 177(^177^Lu)-dotatate in several studies. A phase II randomized parallel assignment study explored the addition of capecitabine plus temozolomide plus PRRT vs. capecitabine plus temozolomide in patients with grade 1/2 pancreatic NETs and capecitabine plus temozolomide plus PRRT vs. PRRT in patients with grade 1/2 midgut NETs [84]. A total of 75 patients were included in the study, and the majority of patients received the treatment in the first- or second-line settings (93.3%). Patients in the combination arms received capecitabine plus temozolomide on its standard D1-D14 dosing schedule, while ^177^Lu-dotatate (7.8 giga-becquerels (Gbq)) was administered IV on D10 every 2 months. In patients with pancreatic NETs, the patients experienced a 12-month PFS of 76 and 67% in the combination and chemotherapy monotherapy arms, respectively. The ORR in the combination and chemotherapy monotherapy arms was 68 and 33%, respectively. The rate of treatment-related grade 3/4 adverse events was not significantly different between the arms (44% in the combination arm and 33% in the chemotherapy alone arm). In the patients with midgut NETs, the patients experienced a 15-month PFS of 90 and 92% in the combination and PRRT monotherapy arms, respectively. The ORR in the combination and PRRT monotherapy arms was 31 and 15%, respectively. The rate of treatment-related grade 3/4 adverse events were significantly different between the arms (81% in the combination arm and 46% in the PRRT monotherapy arm). Longer-term follow-up is needed to see if any OS differences emerge between the arms; however, the ORR in pancreatic NETs is quite striking and may represent a strategy for patients with bulky disease.

A phase II single-arm study combined capecitabine plus ^177^Lu-dotatate in patients with well-differentiated NETs [79]. In this study, 33 patients with advanced disease received ^177^Lu-dotatate 7.8 Gbq IV D1 plus capecitabine 1650 mg/m^2^ PO QD D1-D14 every 8 weeks. Of the 33 patients, 13 possessed small intestinal NETs, 8 possessed pancreatic NETs, and 12 patients possessed primary tumors of unknown origin. The majority of the patients (69.7%) received one line of prior systemic therapy. The median PFS and OS for the patients were not reached at the time of publication. The 12-month and 24-month PFS for the treated patients was 91% and 88%, respectively.

Another phase II single-arm study combined capecitabine plus temozolomide plus ^177^Lu-dotatate in patients with well-differentiated NETs [80]. Of the 35 included patients, 50% possessed tumors of gastroenteropancreatic origin. The patients received ^177^Lu-dotatate 7.8 Gbq IV D1 plus capecitabine 1500 mg/m^2^ PO QD D1-D14 plus temozolomide 200 mg/m^2^ PO QD D10-D14 every 8 weeks. The ORR was 53% (15% complete response rate), the median PFS was 31 months, and the median OS was not reached. Neutropenia (6%), angina (6%), and nausea/vomiting (3%) were the only grade 3 adverse events reported. The long-term safety of this combination was recently reported with a 7-year update from the study [85]. A total of 16% of patients experienced persistent hematotoxicity (defined as a sustained grade ≥ 3 hematologic adverse event beyond the 3-year follow-up), and 8% of the patients developed MDS or acute myeloid leukemia (AML). The estimated cumulative incidence of MDS or AML was 11% in this patient cohort.

A recent retrospective study assessed the activity of capecitabine plus ^177^Lu-dotatate in the first-line setting in patients with grade 1/2 gastroenteropancreatic NETs [86]. In this study, 76 patients either received therapy with ^177^Lu-dotatate (6.0–7.4 Gbq IV D1) plus capecitabine (1250 mg/m^2^ PO QD D1-D14) every 8 weeks or octreotide (30 mg intramuscularly (IM)) monthly. The patients receiving the combination therapy exhibited improved ORR (38% vs. 15%, *p* = 0.025) and DCR (88% vs. 67%, *p* = 0.025) compared to the patients in the octreotide arm. The median PFS in the combination therapy-treated patients was significantly longer than the median PFS in the patients who received octreotide (54 months vs. 16 months, *p* = 0.017). The patients in the combination arm experienced slightly more grade 3/4 hematologic adverse events (anemia 3% vs. 0%, *p* < 0.001; leukopenia 3% vs. 0%, *p* < 0.001) compared to the patients who received octreotide.

From the presented data above, it is not yet evident in which circumstances chemotherapy plus PRRT should be utilized for patients. Relatively high rates of hematotoxicity, particularly MDS, suggest that this approach may be most appropriate for patients with higher-grade or rapidly proliferative tumors that express high levels of somatostatin receptors. Further long-term follow-ups from ongoing combination chemotherapy plus PRRT studies need to be reported to truly define the safety profile of such an approach.

### 5.2. Chemotherapy plus Targeted Therapies

Several studies are exploring the combination of targeted therapies, such as tyrosine kinase inhibitors, and mTOR pathway inhibitors, with chemotherapy. A phase I/II study explored the combination of temozolomide plus pazopanib in 28 patients with pretreated pancreatic NETs [81]. The patients in this study received temozolomide 75–150 mg/m^2^ PO D1-D7 and D15-D21 and pazopanib 400 mg PO daily; the recommended phase 2 dose (RP2D) was temozolomide 75 mg/m^2^ and pazopanib 400 mg. The ORR and DCR were 25% and 70%, respectively. The median PFS and OS were 12.1 months and 36.2 months, respectively. The most common treatment-related adverse events were hepatic toxicity (16%), nausea (5%), and fatigue (5%).

Another phase I/II study explored the combination of temozolomide plus everolimus in patients with pancreatic NETs [82]. The study involved two patient cohorts, one treated with everolimus 5 mg PO daily and temozolomide 150 mg/m^2^ PO D1-D7 and D15-D21 and the other treated with everolimus 10 mg PO daily and temozolomide at the same dosing schedule. A total of 43 patients were enrolled in the study; 77% of the patients were treated in the first-line setting. The ORR and DCR were 40% and 93%, respectively. The median PFS and OS were 15.4 months and NR, respectively. The most common grade 3/4 adverse events were lymphopenia, hyperglycemia, transaminase elevations, thrombocytopenia, and neutropenia, which were observed in 33, 19, 16, 16, and 7%, respectively. The future drug development plans for each of these combinations remain undefined.

### 5.3. Chemotherapy plus Immunotherapy

A multi-cohort phase II trial explored the antitumor activity of the combination of temozolomide with the checkpoint inhibitor nivolumab [83]. The possible synergy between the agents stems from the ability of temozolomide to increase the activation of antigen-presenting cells. The patients in the trial were treated with nivolumab 480 mg IV D1 and temozolomide 150 mg/m^2^ PO D1-D5 every 28 days. Of the 12 patients in the well-differentiated NET cohort, the ORR and DCR were 25 and 92%, respectively. The three patients who achieved a partial response all possessed NETs with Ki-67 indexes ≥15%; two patients possessed grade 3 NETs. The study is ongoing, and the final results may inform the future development plan for the combination.

Given the previous negative data for immunotherapy in patients with well-differentiated neuroendocrine tumors, it remains to be seen whether chemoimmunotherapy combinations will continue to be tested in patients with low-grade, well-differentiated NETs or if the focus will be on high-grade NETs and poorly differentiated NECs.

## 6. Conclusions

The role of chemotherapy in patients with well-differentiated NETs remains an area of active clinical investigation. Patients with pancreatic NETs and grade 3 NETs appear to be the most chemosensitive; however, patients with ≥grade 2 extra-pancreatic NETs may also derive benefits from the treatment modality. In patients with pancreatic NETs, the optimal sequencing of chemotherapy in relation to other available therapies is still an area of ongoing investigation. The SEQTOR trial, for example, is exploring whether alkylating agent-based therapy should come before or after everolimus. A soon-to-be-recruiting clinical trial will explore whether capecitabine plus temozolomide should be offered before or after PRRT. Among the extra-pancreatic primary sites, lung NETs appear to be particularly sensitive to capecitabine plus temozolomide, whereas evidence supporting chemotherapy in midgut NETs is almost absent. Thus far, chemotherapy has demonstrated clinically meaningful antitumor activity in patients with advanced (metastatic or unresectable) disease; its role in adjuvant or neoadjuvant settings is as-of-yet undefined. Temozolomide- and oxaliplatin-based regimens are the most commonly utilized chemotherapies; however, combinations of these regimens with PRRT and targeted therapy are being explored to increase the number of cytoreductive options that may be available for patients with NETs.

## Figures and Tables

**Table 1:** A summary of prospective studies with streptozocin in patients with extra-pancreatic NETs.

Study	Phase	Regimen	N	Key Findings *
Moertel et al. [18]	NS	STZ + 5-FU	42	ORR 33%
		Cyclo + 5-FU	47	ORR 33%
Engstrom et al. [19]	II/III	STZ + 5-FU	80	ORR 22%, OS 16 m
		DOX	81	ORR 21%, OS 12 m
Bukowski et al. [20]	II	STZ + DOX + 5-FU + cyclo	56	ORR 31%
		STZ + 5-FU + cyclo	9	ORR 22%, OS 10.8 m
Sun et al. [21]	II/III	STZ + 5-FU	27	ORR 16%, OS 24.3 m
		DOX + 5-FU	25	ORR 15.9%, OS 15.7 m
Dahan et al. [22]	III	STZ + 5-FU	64 **	PFS 5.5 m, OS 30.4 m
		Interferon α		PFS 14.1 m, OS 44 m

* All times to event endpoints are reported as median values unless otherwise stated. ORR was not uniformly evaluated by RECIST criteria in these studies. ** A total of 6 patients in this trial possessed pancreatic NETs. None of the PFS and OS differences between the arms were statistically significant. Abbreviations: STZ, streptozocin; 5-FU, fluorouracil; cyclo, cyclophosphamide; DOX, doxorubicin; ORR, overall response rate; m, months; OS, overall survival; PFS, progression-free survival; NS, not specified; +, plus.

**Table 2:** A summary of retrospective studies in patients with extra-pancreatic NETs treated with capecitabine plus temozolomide.

Study	N	PTO of Patients Included	Tumor Grades Included	Median CAPTEM Exposure	Key Findings *
Thomas et al. [40]	69	Mixed	G1,G2 and G3	9.5 cycles	9% ORR, 71% DCR, PFS 11 m, OS 38 m
Crespo et al. [41]	19	Mixed	G1 and G2	8 cycles	36.8% ORR, 94.7% DCR, PFS 15.3 m, OS 41.6 m
Chatzellis et al. [42]	49	Mixed	G1,G2 and G3	12.1 cycles	16.3% ORR, 53% DCR, PFS of GI NETs 6.4 m, OS of GI NETs 13.4 m, PFS of lung/thymic NETs NR, OS of lung/thymic NETs NR
Al-Toubah et al. [36]	132	Mixed	G1, G2 and G3	8 cycles	31.8% ORR, PFS 10 m, OS 28 m
Al-Toubah et al. [43]	20	Lung Only	70% TC, 25% AC, 5% NOS	7.5 months	30% ORR, 85% DCR, PFS 14, OS 68 m
Papaxoinis et al. [44]	33	Lung Only	61% AC, 30% TC, 9% NOS	6 cycles	18.2% ORR, 75.8% DCR, PFS 9 m, OS 30.4 months
Chauhan et al. [45]	12	Unknown Primary	G1,G2 and G3	6 cycles	PFS of G2 NETs 10.8 m, PFS of G3 NETs 7 m

* All times to event endpoints are reported as median values unless otherwise stated. Abbreviations: PTO, primary tumor origin; ORR, objective response rate; DCR, disease control rate; PFS, progression-free survival; OS, overall survival; CAPTEM, capecitabine plus temozolomide; TC, typical carcinoid; AC, atypical carcinoid; m, months; G, grade; NR, not reached.

**Table 3:** Prospective trials combining chemotherapy with other systemic therapies in patients with NETs.

Study	Phase	N	Regimen	Key Findings *
Pavlakis et al. [79]	II	75	CAPTEM + ^177^Lu-dotatate (Pancreatic NETs)	12 m-PFS 76%, ORR 68%
			CAPTEM (Pancreatic NETs)	12 m-PFS 67%, ORR 33%
			CAPTEM + ^177^Lu-dotatate (Midgut NETs)	15 m-PFS 90%, ORR 31%
			^177^Lu-dotatate (Midgut NETs)	15 m-PFS 92%, ORR 15%
Claringbold et al. [80]	II	33	Capecitabine + ^177^Lu-dotatate	12 m-PFS 91%, 24 m-PFS 88%
Bhave et al. [81]	I/II	28	Temozolomide + Pazopanib (Pancreatic NETs)	ORR 25%, DCR 70%, PFS 12.1 m and OS 36.2 m
Chan et al. [82]	I/II	43	Temozolomide + Everolimus	ORR 40%, DCR 93%, PFS 15.4 m and OS NR
Owen et al. [83]	II	12	Temozolomide + Nivolumab	ORR 25%, DCR 92%

* All times to event endpoints are reported as median values unless otherwise stated. Abbreviations: PFS, progression-free survival; OS, overall survival; ORR, objective response rate; DCR, disease control rate; m, months; +, plus.

## References

[1] Dasari A., Shen C., Halperin D., Zhao B., Zhou S., Xu Y., Shih T., Yao J.C. (2017). Trends in the Incidence, Prevalence, and Survival Outcomes in Patients With Neuroendocrine Tumors in the United States. JAMA Oncol..

[2] Das S., Dasari A. (2021). Epidemiology, Incidence, and Prevalence of Neuroendocrine Neoplasms: Are There Global Differences?. Curr. Oncol. Rep..

[3] Dasari A., Mehta K., Byers L.A., Sorbye H., Yao J.C. (2018). Comparative study of lung and extrapulmonary poorly differentiated neuroendocrine carcinomas: A SEER database analysis of 162,983 cases. Cancer.

[4] Yao J.C., Pavel M., Lombard-Bohas C., Van Cutsem E., Lam D., Kunz T., Brandt U., Capdevila J., De Vries E.G.E., Tomassetti P. (2015). Everolimus (EVE) for the treatment of advanced pancreatic neuroendocrine tumors (pNET): Final overall survival (OS) Results of a randomized, double-blind, placebo (PBO)-controlled, multicenter phase 3 trial (RADIANT-3). Pancreas.

[5] Yao J.C., Fazio N., Singh S., Buzzoni R., Carnaghi C., Wolin E., Tomasek J., Raderer M., Lahner H., Voi M. (2016). Everolimus for the treatment of advanced, non-functional neuroendocrine tumours of the lung or gastrointestinal tract (RADIANT-4): A randomised, placebo-controlled, phase 3 study. Lancet.

[6] Strosberg J., El-Haddad G., Wolin E., Hendifar A., Yao J., Chasen B., Mittra E., Kunz P.L., Kulke M.H., Jacene H. (2017). Phase 3 Trial of 177Lu-Dotatate for Midgut Neuroendocrine Tumors. N. Engl. J. Med..

[7] Raymond E., Dahan L., Raoul J.L., Bang Y.J., Borbath I., Lombard-Bohas C., Valle J., Metrakos P., Smith D., Vinik A. (2011). Sunitinib malate for the treatment of pancreatic neuroendocrine tumors. N. Engl. J. Med..

[8] Rinke A., Müller H.H., Schade-Brittinger C., Klose K.J., Barth P., Wied M., Mayer C., Aminossadati B., Pape U.F., Bläker M. (2009). Placebo-controlled, double-blind, prospective, randomized study on the effect of octreotide LAR in the control of tumor growth in patients with metastatic neuroendocrine midgut tumors: A report from the PROMID Study Group. J. Clin. Oncol..

[9] Caplin M.E., Pavel M., Ćwikła J.B., Phan A.T., Raderer M., Sedláčková E., Cadiot G., Wolin E.M., Capdevila J., Wall L. (2014). Lanreotide in metastatic enteropancreatic neuroendocrine tumors. N. Engl. J. Med..

[10] Bagnyukova T.V., Serebriiskii I.G., Zhou Y., Hopper-Borge E.A., Golemis E.A., Astsaturov I. (2010). Chemotherapy and signaling: How can targeted therapies supercharge cytotoxic agents?. Cancer Biol. Ther..

[11] Sorbye H., Welin S., Langer S.W., Vestermark L.W., Holt N., Osterlund P., Dueland S., Hofsli E., Guren M.G., Ohrling K. (2013). Predictive and prognostic factors for treatment and survival in 305 patients with advanced gastrointestinal neuroendocrine carcinoma (WHO G3): The NORDIC NEC study. Ann. Oncol..

[12] Yamaguchi T., Machida N., Morizane C., Kasuga A., Takahashi H., Sudo K., Nishina T., Tobimatsu K., Ishido K., Furuse J. (2014). Multicenter retrospective analysis of systemic chemotherapy for advanced neuroendocrine carcinoma of the digestive system. Cancer Sci..

[13] Mitry E., Baudin E., Ducreux M., Sabourin J.C., Rufie P., Aparicio T., Aparicio T., Lasser P., Elias D., Duvillard P. (1999). Treatment of poorly differentiated neuroendocrine tumours with etoposide and cisplatin. Br. J. Cancer.

[14] Thomas K.E.H., Voros B.A., Boudreaux J.P., Thiagarajan R., Woltering E.A., Ramirez R.A. (2019). Current Treatment Options in Gastroenteropancreatic Neuroendocrine Carcinoma. Oncologist.

[15] Broder L.E., Carter S.K. (1973). Pancreatic islet cell carcinoma. II. Results of therapy with streptozotocin in 52 patients. Ann. Intern. Med..

[16] Moertel C.G., Hanley J.A., Johnson L.A. (1980). Streptozocin alone compared with streptozocin plus fluorouracil in the treatment of advanced islet-cell carcinoma. N. Engl. J. Med..

[17] Moertel C.G., Lefkopoulo M., Lipsitz S., Hahn R.G., Klaassen D. (1992). Streptozocin-doxorubicin, streptozocin-fluorouracil or chlorozotocin in the treatment of advanced islet-cell carcinoma. N. Engl. J. Med..

[18] Moertel C.G., Hanley J.A. (1979). Combination chemotherapy trials in metastatic carcinoid tumor and the malignant carcinoid syndrome. Cancer Clin. Trials.

[19] Engstrom P.F., Lavin P.T., Moertel C.G., Folsch E., Douglass H.O. (1984). Streptozocin plus fluorouracil versus doxorubicin therapy for metastatic carcinoid tumor. J. Clin. Oncol..

[20] Bukowski R.M., Johnson K.G., Peterson R.F., Stephens R.L., Rivkin S.E., Neilan B., Costanzi J.H. (1987). A phase II trial of combination chemotherapy in patients with metastatic carcinoid tumors. A Southwest Oncology Group Study. Cancer.

[21] Sun W., Lipsitz S., Catalano P., Mailliard J.A., Haller D.G., Eastern Cooperative Oncology G. (2005). Phase II/III study of doxorubicin with fluorouracil compared with streptozocin with fluorouracil or dacarbazine in the treatment of advanced carcinoid tumors: Eastern Cooperative Oncology Group Study E1281. J. Clin. Oncol..

[22] Dahan L., Bonnetain F., Rougier P., Raoul J.L., Gamelin E., Mitry E., Smith D., Cvitkovic F., Coudert B., Ricard F. (2009). Phase III trial of chemotherapy using 5-fluorouracil and streptozotocin compared with interferon alpha for advanced carcinoid tumors: FNCLCC-FFCD 9710. Endocr. Relat. Cancer.

[23] Kessinger A., Foley J.F., Lemon H.M. (1977). Use of DTIC in the malignant carcinoid syndrome. Cancer Treat. Rep..

[24] Altimari A.F., Badrinath K., Reisel H.J., Prinz R.A. (1987). DTIC therapy in patients with malignant intra-abdominal neuroendocrine tumors. Surgery.

[25] Ramanathan R.K., Cnaan A., Hahn R.G., Carbone P.P., Haller D.G. (2001). Phase II trial of dacarbazine (DTIC) in advanced pancreatic islet cell carcinoma. Study of the Eastern Cooperative Oncology Group-E6282. Ann. Oncol..

[26] Mueller D., Krug S., Majumder M., Rinke A., Gress T.M. (2016). Low dose DTIC is effective and safe in pretreated patients with well differentiated neuroendocrine tumors. BMC Cancer.

[27] Middleton M.R., Grob J.J., Aaronson N., Fierlbeck G., Tilgen W., Seiter S., Gore M., Aamdal S., Cebon J., Coates A. (2000). Randomized phase III study of temozolomide versus dacarbazine in the treatment of patients with advanced metastatic malignant melanoma. J. Clin. Oncol..

[28] de Mestier L., Walter T., Brixi H., Evrard C., Legoux J.L., de Boissieu P., Hentic O., Cros J., Hammel P., Tougeron D. (2019). Comparison of Temozolomide-Capecitabine to 5-Fluorouracile-Dacarbazine in 247 Patients with Advanced Digestive Neuroendocrine Tumors Using Propensity Score Analyses. Neuroendocrinology.

[29] van Hazel G.A., Rubin J., Moertel C.G. (1983). Treatment of metastatic carcinoid tumor with dactinomycin or dacarbazine. Cancer Treat. Rep..

[30] Ollivier S., Fonck M., Becouarn Y., Brunet R. (1998). Dacarbazine, fluorouracil, and leucovorin in patients with advanced neuroendocrine tumors: A phase II trial. Am. J. Clin. Oncol..

[31] Danson S.J., Middleton M.R. (2001). Temozolomide: A novel oral alkylating agent. Expert Rev. Anticancer Ther..

[32] Ekeblad S., Sundin A., Janson E.T., Welin S., Granberg D., Kindmark H., Dunder K., Kozlovacki G., Orlefors H., Sigurd M. (2007). Temozolomide as monotherapy is effective in treatment of advanced malignant neuroendocrine tumors. Clin. Cancer Res..

[33] Kulke M.H., Stuart K., Enzinger P.C., Ryan D.P., Clark J.W., Muzikansky A., Vincitore M., Michelini A., Fuchs C.S. (2006). Phase II study of temozolomide and thalidomide in patients with metastatic neuroendocrine tumors. J. Clin. Oncol..

[34] Chan J.A., Stuart K., Earle C.C., Clark J.W., Bhargava P., Miksad R., Blaszkowsky L., Enzinger P.C., Meyerhardt J.A., Zheng H. (2012). Prospective study of bevacizumab plus temozolomide in patients with advanced neuroendocrine tumors. J. Clin. Oncol..

[35] Murakami J., Lee Y.J., Kokeguchi S., Tsujigiwa H., Asaumi J., Nagatsuka H., Fukui K., Kuroda M., Tanaka N., Matsubara N. (2007). Depletion of O6-methylguanine-DNA methyltransferase by O6-benzylguanine enhances 5-FU cytotoxicity in colon and oral cancer cell lines. Oncol. Rep..

[36] Al-Toubah T., Pelle E., Valone T., Haider M., Strosberg J.R. (2021). Efficacy and Toxicity Analysis of Capecitabine and Temozolomide in Neuroendocrine Neoplasms. J. Natl. Compr. Cancer Netw..

[37] Strosberg J.R., Fine R.L., Choi J., Nasir A., Coppola D., Chen D.T., Helm J., Kvols L. (2011). First-line chemotherapy with capecitabine and temozolomide in patients with metastatic pancreatic endocrine carcinomas. Cancer.

[38] de Mestier L., Walter T., Evrard C., de Boissieu P., Hentic O., Cros J., Tougeron D., Lombard-Bohas C., Rebours V., Hammel P. (2020). Temozolomide Alone or Combined with Capecitabine for the Treatment of Advanced Pancreatic Neuroendocrine Tumor. Neuroendocrinology.

[39] Kunz P.L., Catalano P.J., Nimeiri H., Fisher G.A., Longacre T.A., Suarez C.J., Yao J.C., Kulke M.H., Hendifar A.E., Shanks J.C. (2018). A randomized study of temozolomide or temozolomide and capecitabine in patients with advanced pancreatic neuroendocrine tumors: A trial of the ECOG-ACRIN cancer research group (E2211). J. Clin. Oncol..

[40] Thomas K., Voros B.A., Meadows-Taylor M., Smeltzer M.P., Griffin R., Boudreaux J.P., Thiagarajan R., Woltering E.A., Ramirez R.A. (2020). Outcomes of Capecitabine and Temozolomide (CAPTEM) in Advanced Neuroendocrine Neoplasms (NENs). Cancers.

[41] Crespo G., Jimenez-Fonseca P., Custodio A., Lopez C., Carmona-Bayonas A., Alonso V., Navarro M., Aller J., Sevilla I., Grande E. (2017). Capecitabine and temozolomide in grade 1/2 neuroendocrine tumors: A Spanish multicenter experience. Future Oncol..

[42] Chatzellis E., Angelousi A., Daskalakis K., Tsoli M., Alexandraki K.I., Wachula E., Meirovitz A., Maimon O., Grozinsky-Glasberg S., Gross D. (2019). Activity and Safety of Standard and Prolonged Capecitabine/Temozolomide Administration in Patients with Advanced Neuroendocrine Neoplasms. Neuroendocrinology.

[43] Al-Toubah T., Morse B., Strosberg J. (2020). Capecitabine and Temozolomide in Advanced Lung Neuroendocrine Neoplasms. Oncologist.

[44] Papaxoinis G., Kordatou Z., McCallum L., Nasralla M., Lamarca A., Backen A., Nonaka D., Mansoor W. (2020). Capecitabine and Temozolomide in Patients with Advanced Pulmonary Carcinoid Tumours. Neuroendocrinology.

[45] Chauhan A., Farooqui Z., Murray L.A., Weiss H.L., War Myint Z., Raajasekar A.K.A., Evers B.M., Arnold S., Anthony L. (2018). Capecitabine and Temozolomide in Neuroendocrine Tumor of Unknown Primary. J. Oncol..

[46] Peixoto R.D., Noonan K.L., Pavlovich P., Kennecke H.F., Lim H.J. (2014). Outcomes of patients treated with capecitabine and temozolamide for advanced pancreatic neuroendocrine tumors (PNETs) and non-PNETs. J. Gastrointest. Oncol..

[47] Liu L., Gerson S.L. (2006). Targeted modulation of MGMT: Clinical implications. Clin. Cancer Res..

[48] Kulke M.H., Hornick J.L., Frauenhoffer C., Hooshmand S., Ryan D.P., Enzinger P.C., Meyerhardt J.A., Clark J.W., Stuart K., Fuchs C.S. (2009). O6-methylguanine DNA methyltransferase deficiency and response to temozolomide-based therapy in patients with neuroendocrine tumors. Clin. Cancer Res..

[49] Campana D., Walter T., Pusceddu S., Gelsomino F., Graillot E., Prinzi N., Spallanzani A., Fiorentino M., Barritault M., Dall’Olio F. (2018). Correlation between MGMT promoter methylation and response to temozolomide-based therapy in neuroendocrine neoplasms: An observational retrospective multicenter study. Endocrine.

[50] Cros J., Hentic O., Rebours V., Zappa M., Gille N., Theou-Anton N., Vernerey D., Maire F., Levy P., Bedossa P. (2016). MGMT expression predicts response to temozolomide in pancreatic neuroendocrine tumors. Endocr. Relat. Cancer.

[51] Cives M., Ghayouri M., Morse B., Brelsford M., Black M., Rizzo A., Meeker A., Strosberg J. (2016). Analysis of potential response predictors to capecitabine/temozolomide in metastatic pancreatic neuroendocrine tumors. Endocr. Relat. Cancer.

[52] Liu I.H., Ford J.M., Kunz P.L. (2016). DNA-repair defects in pancreatic neuroendocrine tumors and potential clinical applications. Cancer Treat. Rev..

[53] McEllin B., Camacho C.V., Mukherjee B., Hahm B., Tomimatsu N., Bachoo R.M., Burma S. (2010). PTEN loss compromises homologous recombination repair in astrocytes: Implications for glioblastoma therapy with temozolomide or poly(ADP-ribose) polymerase inhibitors. Cancer Res..

[54] Mei M., Deng D., Liu T.H., Sang X.T., Lu X., Xiang H.D., Zhou J., Wu H., Yang Y., Chen J. (2009). Clinical implications of microsatellite instability and MLH1 gene inactivation in sporadic insulinomas. J. Clin. Endocrinol. Metab..

[55] House M.G., Herman J.G., Guo M.Z., Hooker C.M., Schulick R.D., Lillemoe K.D., Cameron J.L., Hruban R.H., Maitra A., Yeo C.J. (2003). Aberrant hypermethylation of tumor suppressor genes in pancreatic endocrine neoplasms. Ann. Surg..

[56] Le D.T., Durham J.N., Smith K.N., Wang H., Bartlett B.R., Aulakh L.K., Lu S., Kemberling H., Wilt C., Luber B.S. (2017). Mismatch repair deficiency predicts response of solid tumors to PD-1 blockade. Science.

[57] Kim J.W., Cho S.Y., Chae J., Kim J.W., Kim T.Y., Lee K.W., Oh D.Y., Bang Y.J., Im S.A. (2020). Adjuvant Chemotherapy in Microsatellite Instability-High Gastric Cancer. Cancer Res. Treat..

[58] Al-Toubah T., Morse B., Pelle E., Strosberg J. (2021). Efficacy of FOLFOX in Patients with Aggressive Pancreatic Neuroendocrine Tumors After Prior Capecitabine/Temozolomide. Oncologist.

[59] Oziel-Taieb S., Zemmour C., Raoul J.L., Mineur L., Poizat F., Charrier N., Piana G., Cavaglione G., Niccoli P. (2021). Efficacy of FOLFOX Chemotherapy in Metastatic Enteropancreatic Neuroendocrine Tumors. Anticancer Res..

[60] Girot P., Baudin E., Senellart H., Bouarioua N., Hentic O., Guimbaud R., Walter T., Ferru A., Roquin G., Cadiot G. (2019). Oxaliplatin and 5-fluorouracil (FOLFOX) in advanced well-differentiated digestive neuroendocrine tumors: A multicenter national retrospective study from the French Group of Endocrine Tumors (GTE). J. Clin. Oncol..

[61] Kunz P.L., Balise R.R., Fehrenbacher L., Pan M., Venook A.P., Fisher G.A., Tempero M.A., Ko A.H., Korn W.M., Hwang J. (2016). Oxaliplatin-Fluoropyrimidine Chemotherapy Plus Bevacizumab in Advanced Neuroendocrine Tumors: An Analysis of 2 Phase II Trials. Pancreas.

[62] Nagtegaal I.D., Odze R.D., Klimstra D., Paradis V., Rugge M., Schirmacher P., Washington K.M., Carneiro F., Cree I.A., the WHO Classification of Tumours Editorial Board (2020). The 2019 WHO classification of tumours of the digestive system. Histopathology.

[63] Rindi G., Klimstra D.S., Abedi-Ardekani B., Asa S.L., Bosman F.T., Brambilla E., Busam K.J., de Krijger R.R., Dietel M., El-Naggar A.K. (2018). A common classification framework for neuroendocrine neoplasms: An International Agency for Research on Cancer (IARC) and World Health Organization (WHO) expert consensus proposal. Mod. Pathol..

[64] Coriat R., Walter T., Terris B., Couvelard A., Ruszniewski P. (2016). Gastroenteropancreatic Well-Differentiated Grade 3 Neuroendocrine Tumors: Review and Position Statement. Oncologist.

[65] Walter T., Tougeron D., Baudin E., Le Malicot K., Lecomte T., Malka D., Hentic O., Manfredi S., Bonnet I., Guimbaud R. (2017). Poorly differentiated gastro-entero-pancreatic neuroendocrine carcinomas: Are they really heterogeneous? Insights from the FFCD-GTE national cohort. Eur. J. Cancer.

[66] Heetfeld M., Chougnet C.N., Olsen I.H., Rinke A., Borbath I., Crespo G., Barriuso J., Pavel M., O’Toole D., Walter T. (2015). Characteristics and treatment of patients with G3 gastroenteropancreatic neuroendocrine neoplasms. Endocr. Relat. Cancer.

[67] Sorbye H., Baudin E., Perren A. (2018). The Problem of High-Grade Gastroenteropancreatic Neuroendocrine Neoplasms: Well-Differentiated Neuroendocrine Tumors, Neuroendocrine Carcinomas, and Beyond. Endocrinol. Metab. Clin. N. Am..

[68] Raj N., Valentino E., Capanu M., Tang L.H., Basturk O., Untch B.R., Allen P.J., Klimstra D.S., Reidy-Lagunes D. (2017). Treatment Response and Outcomes of Grade 3 Pancreatic Neuroendocrine Neoplasms Based on Morphology: Well Differentiated Versus Poorly Differentiated. Pancreas.

[69] Rogowski W., Wachula E., Gorzelak A., Lebiedzinska A., Sulzyc-Bielicka V., Izycka-Swieszewska E., Zolnierek J., Kos-Kudla B. (2019). Capecitabine and temozolomide combination for treatment of high-grade, well-differentiated neuroendocrine tumour and poorly-differentiated neuroendocrine carcinoma - retrospective analysis. Endokrynol. Pol..

[70] Chan D., Bergsland E., Chan J.A., Gadgil R., Halfdanarson T.R., Hornbacker K., Kelly V., Kunz P.L., McGarrah W., Raj N.P. (2019). Temozolomide in grade III neuroendocrine neoplasms (G3 NENs): A multicenter retrospective review. J. Clin. Oncol..

[71] Jeong H., Shin J., Jeong J.H., Kim K.P., Hong S.M., Kim Y.I., Ryu J.S., Ryoo B.Y., Yoo C. (2021). Capecitabine plus temozolomide in patients with grade 3 unresectable or metastatic gastroenteropancreatic neuroendocrine neoplasms with Ki-67 index <55%: Single-arm phase II study. ESMO Open.

[72] Apostolidis L., Dal Buono A., Merola E., Jann H., Jaeger D., Wiedenmann B., Winkler E.C., Pavel M. (2020). Multicenter analysis of treatment outcomes for well differentiated grade 3 neuroendocrine tumors (NET G3). J. Clin. Oncol..

[73] Howe J.R., Merchant N.B., Conrad C., Keutgen X.M., Hallet J., Drebin J.A., Minter R.M., Lairmore T.C., Tseng J.F., Zeh H.J. (2020). The North American Neuroendocrine Tumor Society Consensus Paper on the Surgical Management of Pancreatic Neuroendocrine Tumors. Pancreas.

[74] Pavel M., Oberg K., Falconi M., Krenning E.P., Sundin A., Perren A., Berruti A. (2020). Gastroenteropancreatic neuroendocrine neoplasms: ESMO Clinical Practice Guidelines for diagnosis, treatment and follow-up. Ann. Oncol..

[75] Prakash L., Bhosale P., Cloyd J., Kim M., Parker N., Yao J., Dasari A., Halperin D., Aloia T., Lee J.E. (2017). Role of Fluorouracil, Doxorubicin, and Streptozocin Therapy in the Preoperative Treatment of Localized Pancreatic Neuroendocrine Tumors. J. Gastrointest. Surg..

[76] Xie H., Liu J., Yadav S., Keutgen X.M., Hobday T.J., Strosberg J.R., Halfdanarson T.R. (2020). The Role of Perioperative Systemic Therapy in Localized Pancreatic Neuroendocrine Neoplasms. Neuroendocrinology.

[77] Cloyd J.M., Omichi K., Mizuno T., Kawaguchi Y., Tzeng C.D., Conrad C., Chun Y.S., Aloia T.A., Katz M.H.G., Lee J.E. (2018). Preoperative Fluorouracil, Doxorubicin, and Streptozocin for the Treatment of Pancreatic Neuroendocrine Liver Metastases. Ann. Surg. Oncol..

[78] Squires M.H., Worth P.J., Konda B., Shah M.H., Dillhoff M.E., Abdel-Misih S., Norton J.A., Visser B.C., Dua M., Pawlik T.M. (2020). Neoadjuvant Capecitabine/Temozolomide for Locally Advanced or Metastatic Pancreatic Neuroendocrine Tumors. Pancreas.

[79] Claringbold P.G., Brayshaw P.A., Price R.A., Turner J.H. (2011). Phase II study of radiopeptide 177Lu-octreotate and capecitabine therapy of progressive disseminated neuroendocrine tumours. Eur. J. Nucl. Med. Mol. Imaging.

[80] Claringbold P.G., Price R.A., Turner J.H. (2012). Phase I-II study of radiopeptide 177Lu-octreotate in combination with capecitabine and temozolomide in advanced low-grade neuroendocrine tumors. Cancer Biother. Radiopharm..

[81] Bhave M., Kircher S.M., Kalyan A., Berlin J., Mulcahy M.F., Cohen S.J., Denlinger C.S., Chiorean G., Sahai V., Zalupski M. (2018). A phase I/II study of the combination of temozolomide (TM) and pazopanib (PZ) in advanced pancreatic neuroendocrine tumors (PNETs) (NCT01465659). J. Clin. Oncol..

[82] Chan J.A., Blaszkowsky L., Stuart K., Zhu A.X., Allen J., Wadlow R., Ryan D.P., Meyerhardt J., Gonzalez M., Regan E. (2013). A prospective, phase 1/2 study of everolimus and temozolomide in patients with advanced pancreatic neuroendocrine tumor. Cancer.

[83] Owen D.H., Wei L., Goyal A., Zhou Y., Suffren S.A., Jacob R., Pilcher C., Otterson G., Verschraegen C., Shah M.H. (2020). CLO20-054: A Phase 2 Trial of Nivolumab and Temozolomide in Advanced Neuroendocrine Tumors (NETs): Interim Efficacy Analysis. J. Natl. Compr. Cancer Netw..

[84] Pavlakis N., Ransom D.T., Wyld D., Sjoquist K.M., Asher R., Gebski V., Wilson K., Kiberu A.D., Burge M.E., Macdonald W. (2020). Australasian Gastrointestinal Trials Group (AGITG) CONTROL NET Study: Phase II study evaluating the activity of Lu-Octreotate peptide receptor radionuclide therapy (LuTate PRRT) and capecitabine, temozolomide CAPTEM)-First results for pancreas and updated midgut neuroendocrine tumors (pNETS, mNETS). J. Clin. Oncol..

[85] Kesavan M., Grover P., Lam W.S., Claringbold P.G., Turner J.H. (2021). Long-term hematologic toxicity of 177Lu-octreotate-capecitabine-temozolomide therapy of GEPNET. Endocr. Relat. Cancer.

[86] Satapathy S., Mittal B.R., Sood A., Sood A., Kapoor R., Gupta R., Khosla D. (2021). 177Lu-DOTATATE Plus Radiosensitizing Capecitabine Versus Octreotide Long-Acting Release as First-Line Systemic Therapy in Advanced Grade 1 or 2 Gastroenteropancreatic Neuroendocrine Tumors: A Single-Institution Experience. JCO Glob. Oncol..

